# Resource use by and trophic variability of *Armases cinereum* (Crustacea, Brachyura) across human-impacted mangrove transition zones

**DOI:** 10.1371/journal.pone.0212448

**Published:** 2019-02-15

**Authors:** Erin Kiskaddon, Kiley Chernicky, Susan Bell

**Affiliations:** Department of Integrative Biology, University of South Florida, Tampa, FL, United States of America; Universidad de Cádiz, Facultad de Ciencias del Mar y Ambientales, SPAIN

## Abstract

In Florida, resource use patterns by *Armases cinereum* (*Armases*), a highly abundant crab in coastal habitats, may serve as important indicators of habitat condition. Here we investigated feeding patterns of *Armases* in coastal palm scrub forest to intertidal mangrove forest transition zones (transitions) as well as the relationship between habitat disturbance and *Armases’* trophic position across three pairs of geographically separated populations in Tampa FL, USA. Each pair of sites represented an unmodified “natural” location as well as a “disturbed” location lacking upland terrestrial palm scrub forested habitat. Laboratory experiments established a baseline understanding of feeding preference of *Armases* offered strictly mangrove material as well as sources abundant at the transition. *In-situ* feeding behavior was examined using MixSIAR mixing models with δ^13^C and δ^15^N stable isotope tracers. *Armases* showed a strong preference for consuming partially-decomposed mangrove material from *Avicennia germinans* and an equally strong preference for *Iva frutescens*. *Armases* also displayed predatory behavior under laboratory conditions, confirming omnivory in the presence of mangrove material. Stable isotopes revealed a pattern of elevated trophic position of *Armases* in disturbed habitats over paired natural locations. Diet reconstruction provided coarse resolution of *in-situ* feeding and results show high spatial variation: in natural habitats, *Armases* appears to rely heavily upon upland plant material compared to disturbed habitats where it may consume more animal prey. Combined, these findings support that *Armases* trophic position and diet may indicate habitat quality in mangrove transitions in the southeastern United States.

## Introduction

Coastal wetlands broadly characterized by emergent vegetation, either as salt marsh-grass dominated, mangrove-dominated, or a mixture of the two vegetation types, support ecosystem functions and provide key ecosystem services [1,2]; these habitats also serve as critical transition zones linking terrestrial and aquatic systems [3–5]. Within the southeastern United States and Gulf of Mexico substantial and sustained reorganization of these ecosystems is occurring [6–7]. While more gradual climate-driven regime shifts in coastal habitats are comparatively well-studied globally [8–9], the ecological implications of acute, anthropogenically-driven modification of coastal ecosystems are less explored but occurring at a high frequency [10–12].The zone encompassing the seaward edge of terrestrial upland pine scrub forest and intertidal mangrove forest in the southeastern United States represents an area of transition between two distinct types of vegetation and a setting within which food web diversity and nutrient fluxes are highly dynamic [13]. However, along coastlines world-wide, the transitions described above (hereafter referred to as “transitions”) are highly susceptible to modification due to human population expansion. Localized alterations in plant composition or creation of artificial boundaries (i.e. roads, structures), defined here as “disturbance”, have been established as key drivers influencing biotic interactions, habitat quality, and food web resilience [14–16]. For example, shading provided by the habitat structure of mangrove canopies influences activities of highly abundant crab taxa as plant cover provides stabilizing effects on temperature fluctuations, regulation of sediment moisture content, and influences growth of microphytobenthos [17,18]. Removal or alteration of mangrove systems can, in turn, have cascading implications for the coupling between landscape and trophic functioning [19,20]. Anthropogenic disturbance leading to mangrove habitat fragmentation has garnered previous attention as it has been linked to significant negative responses by some faunal groups (e.g. herbivorous crabs and mollusks) including localized decreases in biodiversity and biomass [21].

Impacts of human expansion on mangrove habitats remain poorly examined within the subtropical and tropical coastlines of USA. Although extensive effort has been directed to examine the dynamics of food webs and resilience of communities to disturbance in salt marsh grass (*Spartina alterniflora*) habitat in the southeastern United States [11,22], markedly less information is available for native mangrove forests characterized by the taxa *Avicennia germinans*, *Laguncularia racemosa*, and *Rhizophora mangle* (black, white, and red mangroves respectively) [23]. Many studies of mangrove ecosystems have focused upon their role as nursery habitats [24] or the organic matter exchange between mangrove and adjacent marine habitats [25], largely ignoring the interplay between mangrove forest and upland terrestrial habitats. Thus, the ecological implications of modification of upland forest/intertidal mangrove transitions are poorly known, but potentially important in locations with extensive human alterations.

This study addresses the impact of human-generated disturbance on feeding behavior of the conspicuous faunal taxon, the square-back marsh crab, *Armases cinereum* (hereafter, *Armases*) at coastal mangrove transition zones in Tampa Bay, FL, USA. *Armases* is a suitable indicator species of anthropogenic habitat alteration given its high abundance recorded across a variety of coastal habitats in the Northern Gulf of Mexico, including coastal marshes, mangroves, and marsh-fringing terrestrial habitats [26]. Recent research reports negative consequences for *Armases* abundances related to areas with disruption of the coastal transition zone due to residential coastal hardening (introduction of bulkheads) and removal of upland forested habitat along the US Georgia coast [27]. In other systems dominated by mangroves, related sesarmid crabs have been identified as potential keystone species with respect to nutrient cycling and are highly responsive to ecosystem change [28–30]. It is possible that *Armases* may also serve similar important roles throughout their range in the United States.

Detailed information on the feeding behavior of *Armases* in salt marsh habitats dominated by *Spartina* spp. indicates that the crab is generally omnivorous and contributes to both marine and terrestrial food webs [31]. Due to this species’ ability to survive far from water (up to 100 m inland from the coast), some even consider this species a garden pest [32]. Moreover, the generalist feeding strategy and high degree of mobility of *Armases* are thought to be critical to its functional role in marsh/terrestrial transitions [33–35]. As has been reported for crabs in other coastal settings (e.g. *Ocypode* [36]; *Sesarmidae* [37]), *Armases* serves as a “mobile link organism,” (*sensu* [34,35]) contributing to ecosystem development and resilience.

To investigate how disturbance caused by urbanization of mangrove fringe habitat alters resource use patterns of a highly mobile supratidal taxon, we studied *Armases* resource use across mangrove to terrestrial forest transitions. Using a valuable combination of controlled laboratory feeding experiments and *in-situ* δ^15^N and δ^13^C stable isotope mixing models, *Armases* trophic position and diet were evaluated across sites with varying degrees of human disturbance of habitat [38]. Based upon previous investigations into the biology of *Armases* in the saltmarsh/upland forest ecotone [26,31,35], we hypothesized that crabs in disturbed coastal transition zones, characterized by removal of native upland pine scrub forest adjacent to a fringing intertidal mangrove, would display different patterns of resource use compared to crabs in habitats with intact coastal transition zones.

To investigate this hypothesis, we established two main objectives:

1. establish a baseline of *Armases* feeding preferences of mangrove-derived material and examine feeding preferences of *Armases* for common prey taxa abundant at the mangrove/upland transition; and
2. utilize nitrogen (δ^15^N) and carbon (δ^13^C) stable isotopes to reconstruct the diets of *Armases* and compare diets between crabs from disturbed and natural habitats.

Information provided here offers new insight into the behavior of an important consumer in North American coastal mangrove transition habitats with implications for using *Armases* resource use patterns to characterizes anthropogenic impacts on coastal food webs.

## Methods

### Ethics statement

Animal collections were carried out in strict accordance with the Special Activity License issued by the Florida Fish and Wildlife Conservation Commission (License Number: SAL-14-1633-SR) issued 12/05/2014. Additional authorization to conduct field work at the Upper Tampa Bay Regional Park and R.E. Olds Park was obtained from park managers Brian T. Evarts and Lynn Rives, respectively. No protected species were sampled.

### Site description

**Fig 1:**
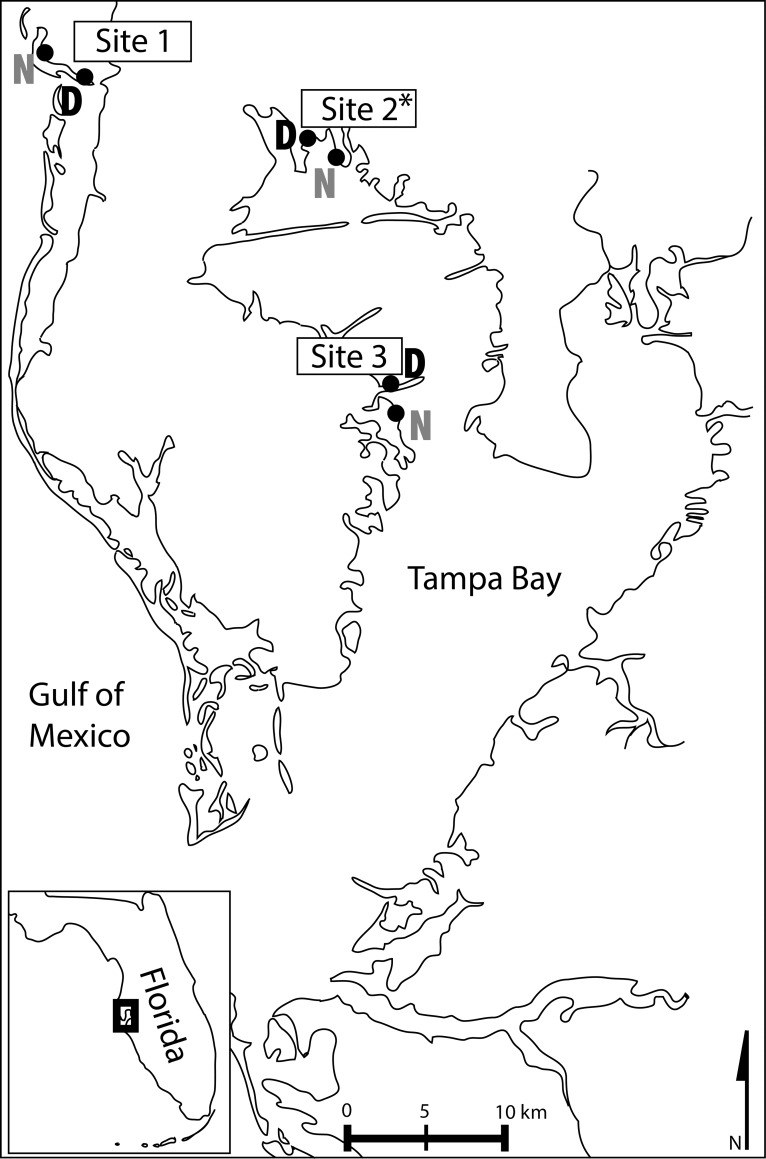
Locations of sampling sites in Tampa Bay, FL. Each site contains one disturbed (D) and one natural location (N) respectively. The asterisk indicates the site at which crabs were collected for the feeding experiments.

Field locations used to assess *in situ* feeding patterns of *Armases* were located at three replicate sites within Tampa Bay, Florida USA (Fig 1). Each site was comprised of two geographically-paired locations, one “undisturbed” (hereafter referred to as “natural”) and one “disturbed”. Each sampling location was a mangrove/terrestrial transition zone with varying dimensions of mangrove habitat and upland forest (Table 1). The natural member of each pair at a site had sections of at least 50 m of continuous habitat connectivity (barring small intrusions of saltmarsh or salt pan habitats) between mixed mangrove forest (consisting of mixtures of three local red, black, and white mangrove taxa) and natural upland scrub forest habitats dominated by the taxa *Pinus* sp., *Quercus* sp., *Serenoa repens*, and *Saba etonia*. The disturbed member of each pair was a mangrove fringe clearly disconnected from the upland forest by anthropogenic-modified habitat (manicured lawn of *Stenotaphrum secundatum* with or without scattered *Sabal palmetto*, and in some cases an adjacent roadway).

**Table 1:** Location labels, GPS coordinates, and qualitative land-use descriptions for the sampling sites in Tampa Bay, FL.

Site	Status	Location Name	Location Coordinates	Description
Site 1	Natural	Honeymoon Island State Park	28°04’07.32”N, 82°49’48.21”W	Mixed mangrove fringe with ~30m width, adjacent to palm scrubland. A Florida State park.
Site 1	Disturbed	Honeymoon Island Causeway	28°03’34.97”N, 82°48’50.09”W	Mangrove fringe with ~4m width, adjacent to asphalt road and housing development, manicured grassy shoulder, scattered planted palms.
Site 2	Natural	Upper Tampa Bay Regional State Park	28°00’53.91”N, 82°38’20.3”W	Mixed mangrove fringe with ~20m width, adjacent to low marsh and palm scrubland. A Hillsborough County park.
Site 2	Disturbed	R.E. Olds Park	28°01’51.72”N, 82°40’09.89”W	Mixed mangrove fringe with ~2m width. Public access, recreation infrastructure, manicured lawn adjacent to mangroves. Mangroves trimmed to ~1m height.
Site 3	Natural	Weedon Island Preserve	27°50’55.28”N, 82°36’14.56”W	Mixed mangrove fringe with ~200m width, adjacent to palm scrubland. A Pinellas County park.
Site 3	Disturbed	Gandy Bridge Causeway	27°52’18.47”N, 82°36’29.53”W	Mangrove fringe with ~7m width, adjacent to sandy shoulder and road.

### Food choice experiments

Documenting *Armases* feeding preference on prey available in mangrove habitats was an essential first step in characterizing the crabs’ trophic role in these systems. Two laboratory feeding experiments were conducted in May 2016 to investigate *Armases* feeding preference for mangrove leaf material and common taxa from the upland transition zone. Feeding was measured using Manly’s alpha (α), a commonly-used diet selectivity index [39–41]. Background information detailing mangrove leaf experiments is available in S1 File.

#### Food source selection rationale

*Armases* crabs and their food sources were collected from Site 2N (see Table 1) where crabs were abundant (mean = 20 ± 3 individuals m^-2^) at the mangrove/upland forest transition (Kiskaddon, pers. observ.). The plant sources used in this feeding experiment: fresh leaf material from *Stenotaphrum secundatum* (Grass), *Borrichia frutescens* (*Borrichia*), *Iva frutescens* (*Iva*), *Nephrolepis biserrata* (Fern), and leaf litter from *Avicennia germinans* (*Avicennia*), represented common and abundant food sources at this location. The mangrove species *Avicennia* was chosen as a food source for the experiment based upon the clear preference of *Armases* for partially decomposed material from *Avicennia* revealed through the mangrove food choice experiment (S1 File). *Gryllodes sigillatus* (cricket) was used as an arthropod prey substitute due to difficulties in collecting enough at a consistent size of the same species of live arthropods (arachnids, insects) from the field site. Preliminary observations of *Armases* in laboratory trials confirmed that crabs would consume each type of food source when offered that food type alone, and that feeding preference when offered cricket prey was similar to feeding preference with other local arthropods. Previous studies of *Armases* in *Spartina*-dominated marsh habitats reported a list of consumed plants like that selected for the current study [32,42], however, the experiment here broadens the range of *Armases* feeding preferences to include mangrove material, also.

#### Plant source collection

Vegetation material was collected as fresh, undamaged leaves from Site 2N in the same location from where the crabs were collected. Leaf material from one individual was used for each feeding trial to address possible differences in palatability between individuals of the same species. *Avicennia* leaves were prepared according to the methods outlined in S1 File to obtain partially-decomposed leaves for feeding trials. This method of creating artificially-decayed *Avicennia* leaves allowed us to control for the duration of decay as the leaves were exposed to intertidal conditions at sediment surface at the same location where both the crabs and the leaves were originally collected. Although not measured directly, we do not believe that *Armases* feeding preferences would be altered using this approach. Upon collection, all vegetation was rinsed with de-ionized water, patted dry, bisected into two equal portions along the midrib, and weighed (g) prior to the start of the experiment. One half of each leaf was used for each crab replicate and the other half was used in a crab-free control to account for autogenic changes as per standards for multi-choice feeding experiments [39].

#### Experimental design

A total of 45 crabs of roughly equal carapace size (10–14 mm) were used in the three feeding trials. Each crab was randomly assigned to each trial (n = 8 males, 7 females per trial). Upon collection, crabs were placed into individual containers filled with filtered seawater to a height of 0.25 cm and starved for 24 h. The crabs were then provided with all five food sources simultaneously (without replacement) and allowed to feed for 48 h in individual containers. All containers were kept at ambient room temperature (24–29° C) with a natural photoperiod (12L, 12D). After 48 h, any remaining food material was collected, rinsed in de-ionized water, patted dry, and dried to a constant weight (g) at 65°C. Initial dry mass was calculated from regression of dry and wet weights. Mass consumed was calculated as the difference between the final dry mass and the estimated initial dry mass, corrected for mass lost due to autogenic change from controls.

#### Statistical analysis

Dietary preference, here defined as any deviation from random consumption of prey, was measured using Manly’s α index (also called Chesson’s index [41]), which is derived from basic probability theory. The food prey selection experiment was conducted without replacing food sources during the experiment as the amounts were not held constant [40,42]. This index was used to preserve natural variation in leaf size and shape, thus the precise mass of each prey item offered was not equivalent across crab consumers. The following equation [43] was used to estimate preference with variable and declining prey numbers (substituting prey mass for counts):

$$\alpha_{i}=\log\;p_{i}/\sum_{j=1}^{m}p_{j}$$ (1)

where α_*i*_ = Manly’s α (preference index) for prey type *i*

*p*_*i*_ = Proportion of prey *i* remaining at the end of the experiment (*i = 1*,*2*,*3…m*) = *e*_*i*_ */ n*_*i*_

*p*_*j*_ = Proportion of other prey items present (*j = 1*,*2*,*3…m*) = *e*_*i*_ */ n*_*i*_

*e*_*i*_ = Mass (g) of prey type *i* remaining uneaten at end of experiment

*n*_*i*_ = Initial mass (g) of prey type *i* in experiment

*m* = Total mass (g) of all prey items

As α values increase, preference for the food item does as well, with negative values indicating avoidance.

Manly’s α selectivity was calculated for each plant prey type and averaged across all trial replicates. *Armases* always consumed the cricket prey first and in its entirety before consuming any plant material (see Results), therefore we were unable to calculate α for cricket prey due to mathematical limitations imposed by the index. No significant differences in feeding preference were observed between sexes or between trials, therefore all crab feeding trials were pooled for subsequent analyses (n = 45). Mean α values of plant prey items were compared using the Friedman Rank Sum test and post-hoc Nemenyi Multiple Comparisons tests. All analyses were conducted using R [44].

### Stable isotope analysis

#### Sample collection and preparation

Stable isotope measurements were used to investigate how habitat connectivity patterns influence trophic position major dietary components of *Armases* as a time-integrated field-based measure across the three sites (Fig 1). Stable isotope ratios of δ^13^C (^13^C/^12^C) and δ^15^N (^15^N/^14^N) were analyzed from consumer crabs as well as possible food sources (dominant vegetation, detritus, and conspicuous fauna, i.e. other arthropods and arachnids). Field observations of *Armases* in both disturbed and natural locations indicate that *Armases* is not restricted to exploitation of sources on the benthos, but rather this species utilizes canopy habitats provided by oak, palm, and shrub vegetation and thus likely exploits many prey types (Kiskaddon, pers. observ.). Although sediment organic matter is exploited by *Armases* in marsh habitats [45,46,32] this was not measured here due to our focus on dense emergent vegetation and a sediment surface covered with plant litter. Furthermore, we recognize that the source groups given here for this mobile omnivore: 1) encompass a broad range of potential source taxa such that in some cases the variability is large, and 2) are numerous which is known to lower the accuracy in mixing model diet reconstruction [47]. Details of collected material are in Table 2. All material for trophic analyses was sampled in June 2016 with a minimum of three individuals of each food source collected as replicates and isotope values averaged for standard deviation. All samples were processed according to the methods outlined by [48].

**Table 2:** Source collection methods and source contribution categories (including relevant photosynthetic pathway for vegetation) for stable isotope analyses.

Category (Photosynthetic Pathway)	Source Organisms	Collection Method
Herbivorous Arthropods (NA)	Leafhoppers (Order Hemiptera), bees (Order Hymenoptera), butterflies (Order: Lepidoptera), aphids (Order: Hemiptera)	Collected using a hand net. Fresh frozen and dried. Whole body analyzed.
Omnivorous/Detritivorous/Carnivorous Arthropods (Secondary Arthropods) (NA)	Spiders (Order: Araneae), dragonflies (Order: Odonata), centipedes (Class: Chilopoda), cockroaches (Order: Blattodea), millipedes (Class: Diplopoda), flies (Order: Diptera), *Orchestia* spp. (Order Amphipoda), ants (Order: Hymenoptera),	Collected by hand from sifted detritus. Fresh frozen and dried. Whole body analyzed.
*Uca spp*. (Fiddler Crabs) (NA)	Fiddler crabs (*Uca pugilator*, *Uca theyeri*, *Uca pugnax*, *Uca minax*)	Collected by hand. Muscle tissue from cheliped analyzed.
*Armases* (NA)	*Armases cinereum*	Collected by hand. Muscle tissue from cheliped analyzed.
Detritivorous Gastropods (NA)	*Melampus coffeus*, *Littoraria angulifera*	Collected by hand from detritus. Tissue from muscular foot analyzed.
Mangrove Detritus (C_3_)	Detrital leaf-litter from some or all of the following: *Avicennia germinans*, *Laguncularia racemosa*, *Rhizophora mangle*	Hand collected leaves partially-decomposed from wrack line. Rinsed and dried.
Seagrass Wrack (C_4_/C_3_)	Detritus from *Halodule wrightii* and and/or *Thalassia testudinum*	Hand collected leaves partially-decomposed from wrack line. Rinsed and dried.
High Intertidal Vegetation (C_3_)	Fresh leaves from *Iva frutescens*, *Borrichia frutescens*, *Limonium carolinianum*, *Baccharis angustifolia*, *Baccharis halimifolia*, *Solidago sempervirens*, *Chenopodium album*, *Physalis angustifolia*	Hand collected fresh leaves from host plant. Rinsed and dried.
Intertidal/Upland Grasses (C_4_)	Fresh leaves from *Distichlis spicata*, *Spartina alterniflora*, *Stenotaphrum secundatum*, *Juncus roemarianus*, *Samolus ebracteatus*	Hand collected fresh leaves from host plant. Rinsed and dried.
Upland Plant Detritus (C_3_)	Detrital leaves and bark from *Pinus elliotti*, *Serenoa repens*, *Sabal palmetto*	Hand collected material from sediment surface. Rinsed and dried.
Upland Plants (C_3_)	Fresh leaves from *Quercus virginiana*, *Nephrolepis biserrata*, *Pteridium aquilinum*, *Myrica cerifera*, *Vitis rotundifolia*, *Amilax auriculata*	Hand collected fresh leaves from host plant. Rinsed and dried.

Samples were analyzed at the USFSI Stable Isotope Lab, Department of Geology, University of South Florida, Tampa, with a Costech ECS Elemental Analyzer with a “zero-blank” autosampler connected to a Thermo Fisher Scientific (FINNIGAN) Delta V 3 keV isotope ratio mass spectrometer. The measured stable isotope ratios of ^13^C/^12^C and ^15^N/^14^N are reported as δ^13^C and δ^15^N in ‰ units relative to the standards, Vienna PeeDee Belamnite carbon and air nitrogen, respectively. Standard delta notation was used: δ^13^C or δ^15^N = {(Rsample/Rstandard)-1}*1000, where R is respectively ^13^C/^12^C or ^15^N/^14^N. Percent C and N were used in mixing model analysis to account for concentration dependence. Information related to δ^13^C and δ^15^N signatures of consumers and sources can be found in S2 File.

#### Trophic position

Relative trophic position of *Armases* was determined using δ^15^N values of individual crabs [49–53] and the model outlined by [53]. The Δδ^15^N enrichment value used was +5.2 ± 0.28‰ (± 1 SD) derived from [54], the averaged Δδ^15^N enrichment values of two related sesarmid mangrove crabs, *Episesarma singaporense* and *E*. *versicolor* (Sesarmidae). This averaged value of the two *Episesarma* species was selected for use over values derived from just one of the species assessed in [54] due to *Armases*’ known omnivorous feeding and high uncertainty as to the reliance of *Armases* on mangrove material as a food source. Although species-specific enrichment values are ideal for calculations of both trophic position and diet reconstruction given the high variation in Δδ^15^N and Δδ^13^C [54], calculations of those factors were not included in this study. For further rationale on our use of these enrichment values, see S3 File. To calculate trophic position, the δ^15^N baseline (see [55] and refs. therein) was the δ^15^N signature of *Melampus coffeus*, a common detritivorous snail, found abundant at each sampling site (Kiskaddon, pers. observ.) and which has been confirmed as a sufficient base metric for trophic position analyses [53].

Information collected on trophic position of *Armases* was compared between disturbed and natural habitat types. A nested ANOVA was performed using habitat type as the main fixed effect and the four sites as the random grouping variable. Post-hoc pairwise t-tests were then used to determine whether trophic position differed significantly between the paired habitat types within each site.

#### Mixing models

Mixing models were used to reconstruct diets of *Armases* by linking consumer δ^15^N and δ^13^C isotopic signatures to the signatures of possible dietary sources [56]. However, due to restrictions and known shortcomings of diet reconstruction models (including MixSIAR) regarding large numbers of possible sources and overlapping signatures in isotopic space [57], overlapping stable isotope signatures and species of similar ecology/trophic category were grouped together (Table 2). This method sometimes introduced large standard deviations in source signatures, and our estimates of dietary proportions should therefore be viewed as approximations of true dietary proportions. The Δδ^15^N and Δδ^13^C trophic enrichment factors (+5.2 ± 0.28 and +4.6 ± 0.71 respectively) were selected based on amended values from [54], see rationale above.

While Bayesian mixing models will automatically calculate source contributions even when a model is highly unlikely to satisfy the point-in-polygon assumption for every consumer [58], an *a priori* Monte Carlo simulation of mixing polygons was performed based on the methods outlined by Smith et al. [59] to determine if mixing models were appropriate. The mixing polygon simulation provides iterative mixing polygons which satisfy the point-in-polygon assumption of Bayesian mixing models (e.g. that the stable isotope signatures of all consumers lay within a convex polygon formed by outlining the isotopic signatures of all the sources in isotopic space) [60]. This was used to determine whether the proposed mixing models were likely to explain the isotopic signatures of all consumers at each study site. Mixing model simulations were generated to quantify a 95% confidence mixing region formed by the sources sampled for each site. No *Armases* fell outside of the 95% mixing region indicating that the food items examined can explain the consumers diet.

After mixing regions were confirmed for all sites at 95% confidence, the Bayesian Stable Isotope Mixing Model platform in R (MixSIAR) was used to construct mixing models incorporating concentration dependence to calculate the most likely dietary proportions of the sources examined for each population of *Armases* [61,62]. Using MixSIAR, two fixed effect models were run using site as the covariate amongst either all disturbed or natural areas. Site was chosen as the fixed effect because we were interested in the variation in *Armases* between sites and not in the overall diet. Each model was run with a Markov chain Monte Carlo (MCMC) chain length of 3,000,000 replicates of which the first 1,500,000 were dropped as burn-in to allow the mcmc to reach its equilibrium before collecting data points. After burn-in, subsequent iterations were then stored and checked using convergence diagnostics. The iterations were averaged to produce the most likely proportions with one standard deviation of each source for each *Armases* population. Natural and disturbed populations were measured separately due to unequal sources (the natural sites included upland sources not available to disturbed populations) and mixing model limitations.

## Results

### Feeding choice

Laboratory feeding trials using transition zone food sources indicated that *Armases* readily consumed both plant and animal items. Across all trials, consumption of animal prey (crickets), was overwhelmingly greater than consumption of all other food sources. In fact, cricket prey offered to each *Armases* was invariably consumed in its entirety within 10 minutes and before any other prey items were consumed.

**Fig 2:**
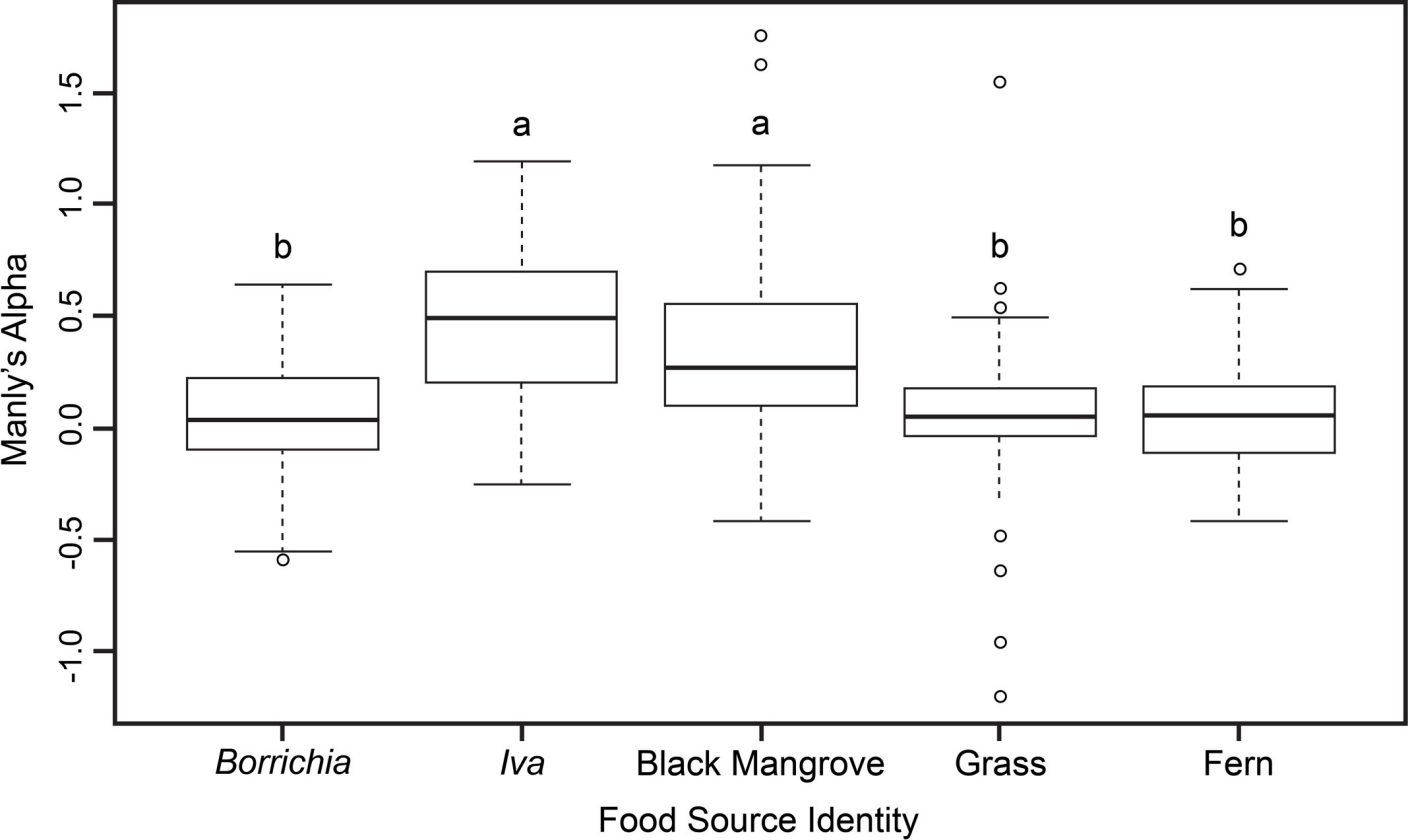
Mean feeding selectivity (Manly’s α feeding selectivity index) for the vegetation prey sources consumed by *Armases* in the transition zone feeding experiment. Scores for each food were averaged across all crab replicates. Letters denote significant differences at α = 0.05 level of significance (Friedman rank sum and post-hoc Nemenyi multiple comparisons tests).

Manly α values (Fig 2) indicated that two plant food sources had higher selectivity indices than others and that the differences in selectivity of food based on plant species identity were significant (Friedman rank sum test: Friedman χ2 = 48.6, p-value = 7.26e^-10^). Further pairwise analyses revealed significantly greater α values (higher selectivity) for *Avicennia* (mean α = 0.38, Nemenyi Multiple Comparisons tests, p = 0.02) and *Iva* (mean α = 0.46, Nemenyi Multiple Comparisons tests, p < 0.01) by crabs compared to the other plant food sources. Mean α selectivity was not significantly different between *Iva* and *Avicennia* (Nemenyi Multiple Comparisons tests, p > 0.05) (Fig 2).

### Stable isotope analysis

#### Trophic position

**Fig 3:**
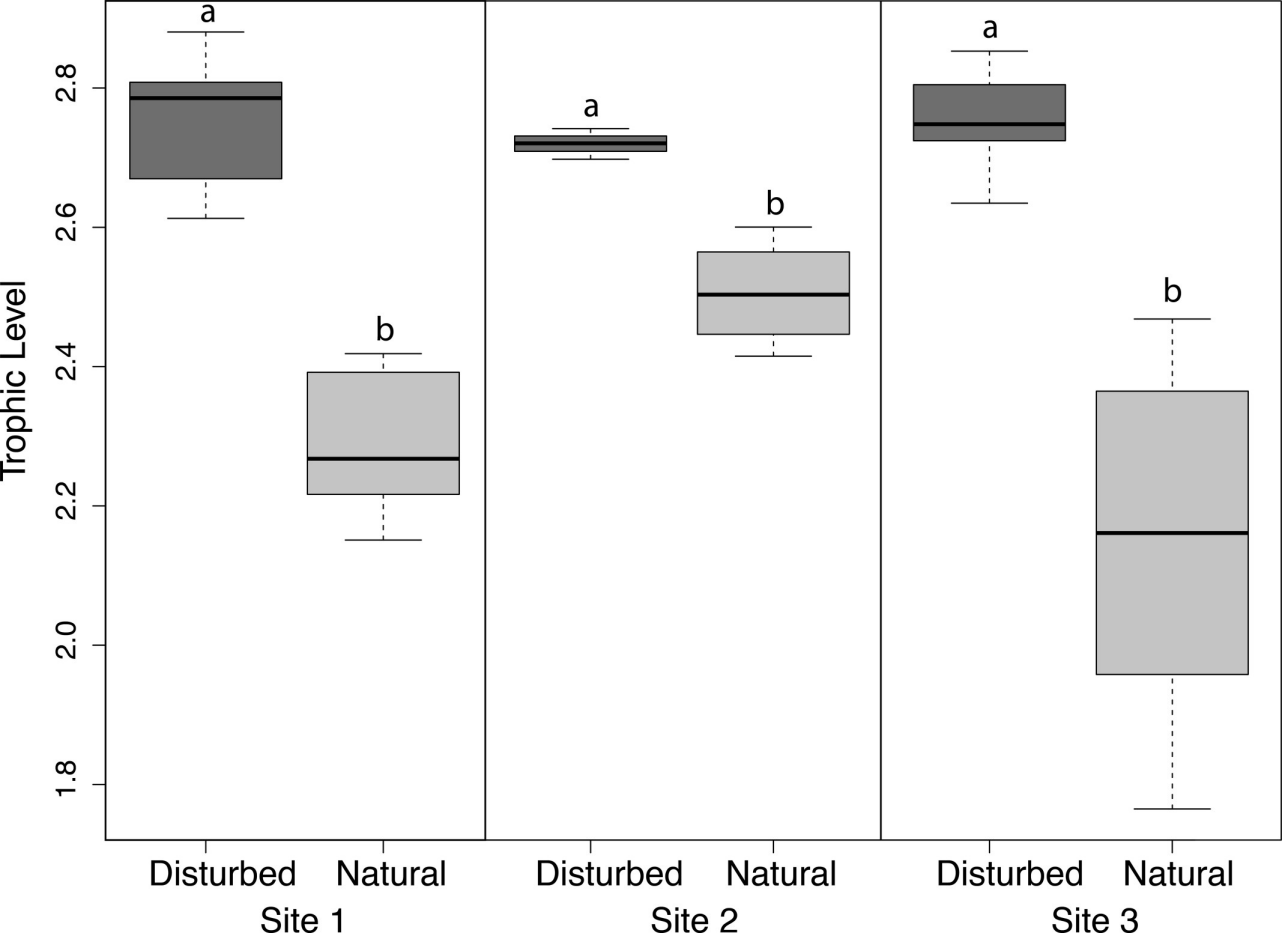
Trophic position of *Armases* based on δ^15^N isotope signatures. Levels are relative to the site-specific baseline, *Melampus coffeus*, sampled at each location. Letters denote significant pair-wise differences between disturbed and natural locations at each site (ANOVA, α = 0.05 level of significance).

*Armases* trophic position, calculated using δ^15^N, revealed a consistent pattern of significantly elevated trophic position at the disturbed location compared to the natural location for each of the three sites (Fig 3). A nested ANOVA indicated that both habitat treatment (disturbed vs. natural) as well as site 1–3 were significant factors influencing trophic position (Table 3). Measured individually, post-hoc pairwise Tukey HSD tests indicated significant differences between the trophic position of crabs from natural and disturbed populations at each site (p<0.05).

**Table 3:** Nested ANOVA comparing *Armases* trophic position between disturbed and natural locations across sites.

Source	Sum of Squares	DF	Mean Square	F value	P
**Treatment (Natural/Disturbed)**	1.63	1	1.63	73.81	6.25e-9
**Site within Treatment (1–3)**	0.31	4	0.08	3.53	0.02
**Residuals**	0.55	25	0.02		

#### Mixing models

**Fig 4:**
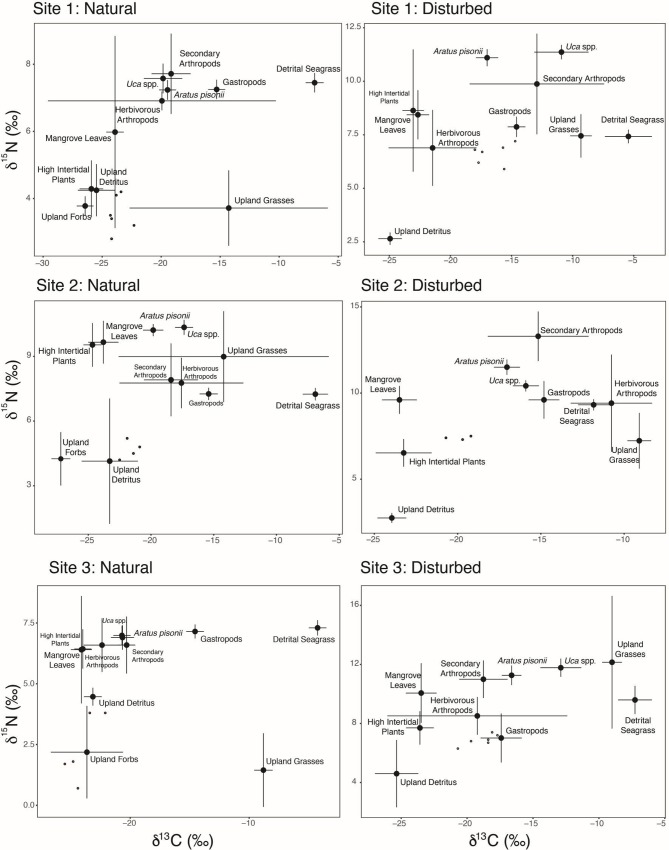
Biplots of all *Armases* δ^13^C and δ^15^N isotope signatures plotted with site-specific source groups (mean ± s.d.).

Bayesian mixing models using δ^13^C and δ^15^N stable isotope tracers and %C and %N concentrations were used to estimate proportions of source (prey) contributions to each of the *Armases* consumer populations examined. The positions of each *Armases* population and the contributing sources in isotopic space were highly variable (Fig 4), highlighting the high degree of spatial variability in both sources and consumers across Tampa Bay. As expected, C_4_ and C_3_ plant sources separated distinctly in isotopic space across sites with grasses exhibiting δ^13^C values at approximately -13‰ and upland forest (including mangrove material) C_3_ plants at ~27‰ (see Table 2 for information on plant sources and photosynthetic pathways). Unsurprisingly the δ^13^C signatures of upland forbs, high intertidal plants, and mangrove sources largely overlapped in isotopic space, however for most sites, mangrove material and high intertidal plants showed elevated δ^15^N signatures relative to other C_3_ plant sources. The higher nitrogen content in detrital mangrove material and high intertidal plants (including *Iva*) may be due to increased microbial activity or inherently higher leaf nitrogen content [63,64]. In some cases, the large standard error for source groups can be attributed to grouping of sources, thus limiting the possible resolution of mixing models to accurately reconstruct *Armases* diets.

**Fig 5:**
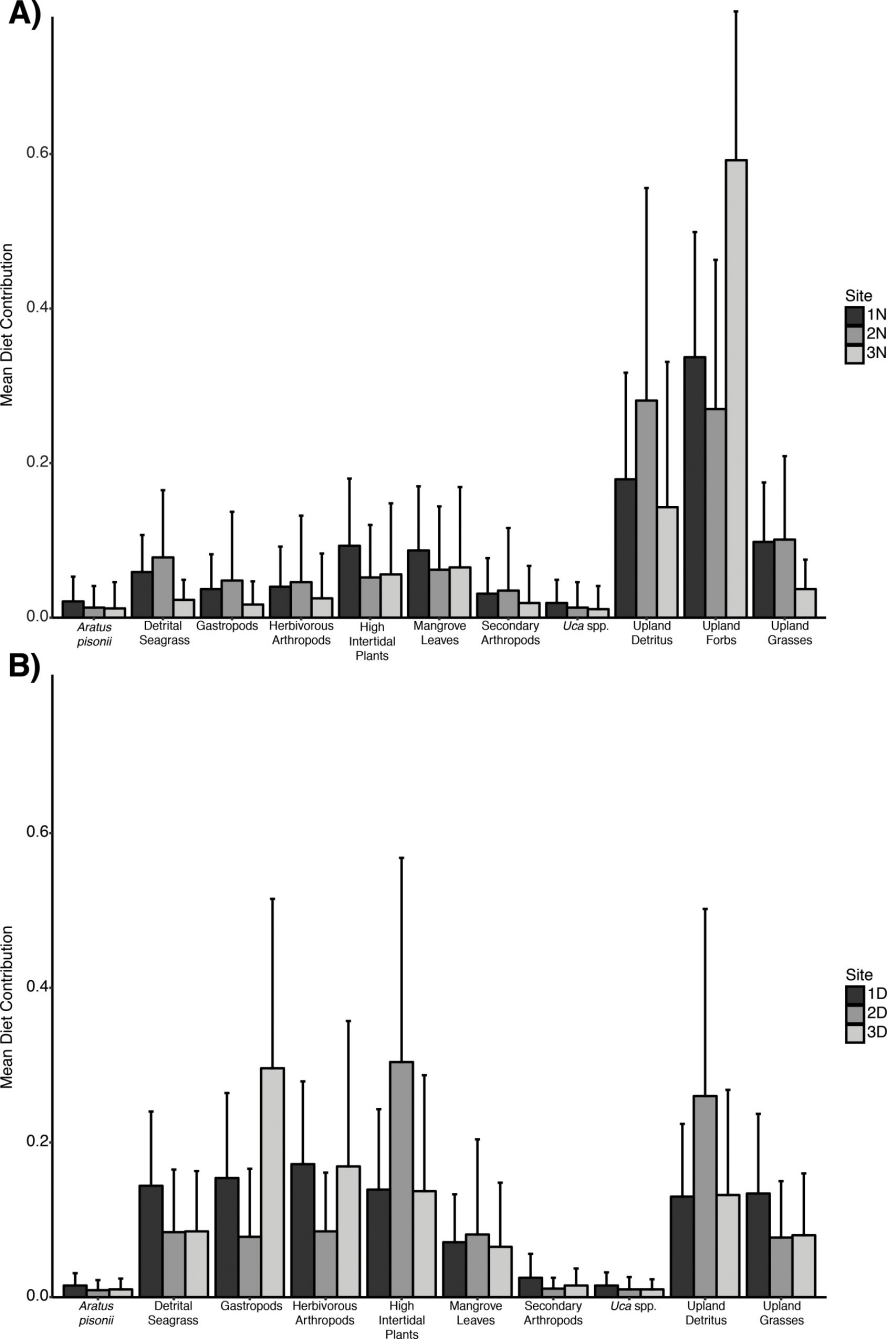
Average source contributions (± SE) derived from separate MixSIAR mixing models for *Armases* in A) natural and B) disturbed sites.

Reconstructed diets, reported as the proportional contribution of measured sources to *Armases* consumers, indicated wide variability. No consistent patterns were observed across individual sites related to mangrove sources, but a larger contribution of both animal sources (gastropods and herbivorous arthropods) and high intertidal plants to the diet of *Armases* sampled from disturbed sites is observed (Fig 5). Surprisingly, carnivorous and detritivorous insects and arachnids (referred to here as “secondary arthropods”) did not appear to contribute a large proportion to the *Armases* diet at any site. Upland forbs, although not present at the disturbed locations (and thus not included in the mixing models for those sites), were estimated to contribute a large proportion to the diet of *Armases* in natural sites.

## Discussion

Mixing model diet reconstruction coupled with controlled laboratory feeding experiments served here as a powerful and useful approach for investigating how habitat condition impacts dietary choices of the crab, *Armases*, at the mangrove/terrestrial transition. Overall, our results indicate that *Armases* inhabiting disturbed transitional habitats have different patterns of resource use compared to those in natural transitions. While *Armases* consumed a variety of food sources too numerous, or some too similar in isotopic signature, to track diet reconstruction with stable isotopes alone, our laboratory experiments convincingly showed that this crab taxon preferentially consumed insects, fresh *Iva* leaves, and partially-decomposed material of *Avicennia*, black mangroves. Combined these findings expand our understanding about *Armases* activities in coastal habitats and provide information related to the trophic position of this species in mangrove habitats. Overall, we identify the functional role of *Armases* as an omnivore, serving as a predator, herbivore and detritivore in mangrove transition systems.

Results from *in-situ* dietary analysis based on stable isotopes, as well as our controlled feeding experiments, provided new insight into the impact of anthropogenic habitat fragmentation on patterns of this species’ resource use. Via stable isotope analysis, we discovered that tissues from *Armases* consumers from disturbed locations reflected higher ^15^N enrichment and higher trophic position, suggesting the primary nitrogen source for these crabs was one of elevated ^15^N [65]. Such a pattern may be indicative of greater consumer reliance on animal prey over plant food sources in disturbed habitats, however more rigorous field assessments of vegetation or animal sources were not conducted to link elevated *Armases* nitrogen directly to higher abundances of potential nitrogen-rich prey. In this study the pattern of elevated nitrogen is supported both by laboratory experiments where *Armases* preferentially consumed the animal prey over vegetation sources as well as by mixing model analysis that clearly indicated greater reliance of *Armases* on gastropod and herbivorous arthropod sources in disturbed over natural sites. Both field observations (Kiskaddon, pers. observ.) and earlier reports indicate that *Armases* consumes invertebrate prey in the field including: spiders, *Uca spp*. (fiddler crabs), *Melampus spp*. and *Littoraria spp*. (intertidal gastropods), *Orchestia spp*. (amphipods), aphids (and other grazing insects), and other various arthropods [31,35,45]. Although our results did not indicate high relative proportions of secondary arthropods to *Armases* diets, the exploitation of herbivorous arthropod prey is supported by [31]. Elevated trophic position linked to increased reliance on animal prey may reflect factors including high abundance/availability of animal sources in crab diets. Given that, visually, disturbed areas were lacking in preferred plant sources (Table 1), the reduced abundance of preferred plant sources, such as *Avicennia* and *Iva*, in disturbed locations compared to that found in natural sites can also explain our recorded differences in trophic position of *Armases*.

Range of trophic position is also informative for comparing resource use patterns of consumers. We observed a narrower range of trophic position in *Armases* from disturbed versus natural habitats- indicative of patterns of less diverse resource use likely driven by a reduced pool of available palatable food sources. Reduced variation in trophic position may have consequences for this species’ resilience and may possibly impact broader food web stability [66,67]. In contrast, the wider range in trophic position observed in collections of *Armases* from natural versus disturbed locations suggests a more diverse pattern of resource use in non-impacted mangrove habitats [65,67]. A diverse feeding regime may reflect *Armases’* feeding advantageously on both abundant, comparatively nutrient-poor food (e.g., plants) and rarer, nutrient-rich foods (e.g. animal prey) [45,68]; this flexibility may allow *Armases* to persist in densities of 20–50 crabs m^-2^ across a variety of coastal systems. Tewfik et al. [69] also noted a similar behavioral pattern in the ghost crab, *Ocypode quadrata*, a generalist consumer in sandy beach habitats. Specifically, as was found for *Armases*, the ghost crab displayed a shift in its diet and trophic position in response to anthropogenically-driven habitat disturbance. More studies are requisite to determine whether shifts in trophic position are a general trend accompanying human-modified boundaries in coastal ecosystems.

Previous experiments conducted by Kristensen et al. [70] reported that controlled laboratory feeding experiments can both synergistically improve accurate identification of dietary components from stable isotope analyses and elaborate on trophic food web structure; this was evident in our study, as well. Although diet reconstruction using mixing models was not able to distinguish among overlapping sources from C_3_ plants (i.e. mangrove, high intertidal, and upland forbs), laboratory experiments provided the needed resolution of detailed feeding preferences of crabs. The results of our mangrove leaf trials are consistent with previous observations that feeding on vegetal food sources is often constrained by tannin content and that *Armases* prefers leached vegetation [35,45]. Tannins are one of many important plant tissue compounds responsible for deterring terrestrial herbivory [71], and mangrove leaves are known to have high tannin content [72]. Here, although we did not measure tannins directly, we observed that *Armases* showed much greater leaf consumption rates in leached, partially-decomposed leaf trials over both fresh and newly-senesced mangrove leaf trials (see S1 File), as well as clear patterns of feeding preference for *Avicennia* over the other mangrove taxa. The low consumption rates in fresh and senescent leaf trials is consistent with previous work related to sesarmid crab feeding in mangrove habitats that show a strong negative correlation between leaf tannin content (highest in fresh leaves) and herbivory rates [71]. Furthermore, preference for *Avicennia* displayed by *Armases* over red and white mangrove taxa in both fresh and partially-decomposed leaf trials indicates that palatability for *Avicennia* extends beyond enhanced microbial activity during the process of decay. *Avicennia* leaves are reported to have higher nitrogen content than other mangrove taxa [63], which is aligned with suggestions that high nitrogen content influences detritivory patterns in other Grapsid crabs [63,73].

Improved resolution of food selectivity of our target consumer emerged from controlled food choice studies within which multiple taxa from the mangrove/upland ecotone were offered simultaneously. *Armases* exhibited clear preferences for cricket animal prey and marked avoidance for Grass, *Borrichia*, and Fern foods when *Iva* and *Avicennia* material was available. The Manly selectivity indices indicated preference of *Armases* for *Iva* and *Avicennia* material over the other plant taxa offered. Previous studies indicate that *Iva* may be less palatable to *Armases* at lower latitudes (i.e., Sapelo Island, GA) versus higher latitudes (i.e, Rhode Island) along the Atlantic coast [74], however we saw no differences in palatability between *Iva* and *Avicennia* plant sources. A similar selectivity for *Avicennia* and *Iva* may also be driven by familiarity because mangrove material is more readily available and encountered more frequently by *Armases* in southern versus northern latitudes. *Armases* likely relies on nitrogen-rich plant species such as *Iva* as shown by others (e.g., [74,64,75]), but our laboratory study revealed an additional reliance of *Armases* on partially-decomposed *Avicennia* and live animal material–food items likely to be encountered at the mangrove/upland transition.

## Implications

Modification of mangrove and marsh habitats via coastal development has been repeatedly cited as an increasing threat to ecosystem connectivity [76]. So, too, may human alteration of coastal landscapes impact landward shift of the mangrove/upland forest boundary due to sea-level rise [6]. Gilman et al. [77] note that many factors influence movement of mangrove fringe habitats and that the presence of obstacles to landward migration (e.g. seawalls, roads) may cause a gradual reduction in overall mangrove habitat. Displacement of saltmarsh and forest vegetation with respect to mangrove encroachment also has been documented to result in ecosystem-level effects [2,78–81]. The results of this study indicate that if mangrove landward expansion displaces upland forested vegetation and human infrastructure further fragments mangrove fringe habitat, then the resulting disruptions to *Armases* feeding behavior may have large-scale implications for subsidizing nutrients to adjacent habitats. Given that *Armases’* diet reflects availability of a variety of prey associated with mangrove, saltmarsh, and upland habitats [31,35], tight coupling between trophic position and spatial arrangement of mangrove and upland forest is expected [21].

Our findings demonstrate that *Armases’* generalist feeding behavior, as well as its interchangeable preference for *Avicennia* mangrove detritus and *Iva* leaf material, may contribute to *Armases’* capacity to persist when coastal transitions are anthropogenically modified or shift along tidal elevation [67]. Prevalence of one or both plant taxa may be an important factor when considering resilience of *Armases* populations to habitat alteration/degradation. Our findings also provide support for McCann et al.’s [82] assertion that *Armases* may be a useful indicator of shifts in trophic structure as human impacts spread across coastal landscapes, potentially restricting access to food sources as well as suitable habitat. Further investigations into the role of *Armases* as a mobile link between habitats (e.g. *in situ* feeding observations, manipulations, and examinations of habitat utilization of intertidal mangrove vs upland terrestrial forested habitats) would be useful for determining the role of this species in mangrove food webs that were not revealed using stable isotopes or controlled laboratory studies.

## Supporting information

S1 File Mangrove feeding experiment. (DOCX)

S2 File Data supplemental. (XLSX)

S3 File Enrichment rationale supplemental. (DOCX)
