# Supplementary material for: Resource use by and trophic variability of Armases cinereum (Crustacea, Brachyura) across human-impacted mangrove transition zones
Source: PLoS One. 2019 Feb 15;14(2):e0212448. doi: 10.1371/journal.pone.0212448 (PMC6377144; doi:10.1371/journal.pone.0212448)
Supplement: S1 File — (DOCX) [file pone.0212448.s001.docx]

**S1 File: Mangrove Feeding Experiment**

To investigate mangrove herbivory in the mangrove / upland forest transition zone, a controlled laboratory mangrove leaf feeding experiment was conducted using both crabs and leaf material from the natural location at Site 2.

**Methods:**

**Leaf Collection:**

We measured the feeding rates of crabs on leaves of three mangrove taxa (*Rhizophora mangle* [Red]*, Laguncularia racemosa* [White]*,* or *Avicennia germinans* [Black]), and each in three different conditions: fresh, senescent, and partially-decomposed. Leaves were collected from three individual trees selected along the same elevation for each of the mangrove species. Fresh leaves were collected by removing undamaged green leaves from one branch to minimize within-tree variation. Senescent leaves were characterized as yellowed leaves which were still attached to the tree and easily removed. Due to the low availability of senescent leaves within a single tree branch, senescent leaves were collected from any branch of the same tree from which the fresh leaves were collected. Partially-decomposed leaves were created by collecting senescent leaves (as described previously) which were then placed in litter bags (28 x 32 cm) of fine mesh (2 x 2 mm) on the sediment surface at mean tide height for 14 days to allow for both rapid and gradual phases of decomposition [1]. The preparation of leaves and experimental design followed the methodology outlined by Ashton et al., [2]. Once collected, each leaf was rinsed, patted dry, divided in half by cutting down the midrib, and refrigerated for 24 h while the crabs were acclimatized to laboratory conditions. After the acclimatization period, each leaf half was weighed (g) prior to the start of an experiment. Half of each leaf was used in an individual crab treatment and the other was used as a “crab-free control” (n = 10 per trial) to account for differences in leaf mass due to leaching.

**Crab Collection:**

As with the ecotonal feeding experiment, the mangrove leaf condition experiment utilized forty-two crabs (21 males, 21 females) of roughly equal carapace size (between 10 and 14 mm) which were hand-collected at the ecotone between mangrove and upland forest habitats in the same area where mangrove leaf collections were conducted. Crabs were randomly divided into three trial groups (n = 14) with equal numbers of males and females. Crabs were held in individual plastic containers and each container was filled with filtered seawater to a height of 0.25 cm and kept at ambient room temperature (24-29° C) and natural photoperiod (12 L/12 D). Crabs were placed into containers and starved for 24 h before the start of each experiment.

**Experimental Design:**

Consumption rates were examined by offering leaves of all three species of mangrove simultaneously to a crab consumer. During the experiment each consumer was provided with a choice of three types of leaf material from each of the three mangrove taxa and allowed to feed freely for 48 h. Control treatments were run simultaneously. At the conclusion of the experiment, any remaining leaf material was removed, rinsed, and dried to a constant weight (g) for 48 h. Initial dry mass was estimated from wet mass via regression and mass consumed was calculated and corrected for the mass lost due to leaching. Results are expressed as the percentage of initial dry mass consumed.

**Results:**

Differential consumption based on crab sex was determined to be insignificant and therefore sex was not a factor in subsequent analyses. No significant differences in crab feeding between trials were found using a Kruskal-Wallis Test, p < 0.05) indicating no differences in leaf palatability among individual trees of the same mangrove species, and thus the three trials for each experiment were pooled (total n = 45 replicates). Results of a Friedman Rank Sum test and the *post-hoc* Nemenyi Multiple Comparisons test indicated that *Armases* consumed a significantly greater proportion (p < 0.05) of Black mangrove leaf mass in fresh and partially-decomposed experiments over the other mangrove taxa offered. No significant differences in feeding were observed when senescent leaves were offered and observed feeding rates were highly variable.

**References**

1. Lima R, Colpo DK. Leaf-litter decomposition of the mangrove species *Avicennia schaueriana, Laguncularia racemosa* and *Rhizophora mangle*. J Mar Biol Assoc UK. 2014; 233-239.
2. Ashton E. Mangrove sesarmid crab feeding experiments in Peninsular Malaysia. J Exp Mar Biol and Ecol. 2002; 273: 97-119.
