# Supplementary material for: Resource use by and trophic variability of Armases cinereum (Crustacea, Brachyura) across human-impacted mangrove transition zones
Source: PLoS One. 2019 Feb 15;14(2):e0212448. doi: 10.1371/journal.pone.0212448 (PMC6377144; doi:10.1371/journal.pone.0212448)
Supplement: S3 File — (DOCX) [file pone.0212448.s003.docx]

**S3 File: Enrichment Rationale Supplemental**

Species- and habitat-specific trophic enrichment factors allow for the most accurate calculations of trophic position and diet reconstruction when using stable isotope inferences [1]. Variations in enrichment factors have been shown to vary by taxon, environment, and tissue [2], however such a factor does not exist for *Armases* in mangrove habitats. Thus, we used enrichment factors proposed by Herbon and Nordhaus [3] to estimate the fractionation of *Armases* from its mangrove food sources. We note that our approach (using an estimated enrichment factor, source groupings, and large numbers of sources) only allows for a coarse approximation of diet reconstruction, but our results clearly indicate that *Armases* displays different feeding behaviors across habitats as a result of habitat modification.

**References**

1. Bond AL, Diamond AW. Recent Bayesian stable-isotope mixing models are highly sensitive to variation in discrimination factors. Ecol Appl. 2011; 21: 1017-1023.
2. Caut S, Angulo E, Courchamp F. Variation in discrimination factors (Δ^15^N and Δ^13^C): the effect of diet isotopic values and applications for diet reconstruction. J Appl Ecol. 2009; 46: 443-453.
3. Herbon CM, Nordhaus I. Experimental determination of stable carbon and nitrogen isotope fractionation between mangrove leaves and crabs. Mar Ecol Prog Ser. 2013; 490: 91-105.
